# Supplementary material for: Classifying patents based on their semantic content
Source: PLoS One. 2017 Apr 26;12(4):e0176310. doi: 10.1371/journal.pone.0176310 (PMC5405987; doi:10.1371/journal.pone.0176310)
Supplement: S1 Text — Describes with more details the definition of patents and context. (PDF) [file pone.0176310.s001.pdf]

S1 Text : Definition of utility patent

A utility patent at the USPTO is a document providing intellectual property and protection of an invention. It excludes others to making, using, or selling the invention the same invention in the United States in exchange for a disclosure of the patent content. The protection is granted for 20 years since 1995 (it was 17 years before that from 1860) starting from the year the patent application was filled, but can be interrupted before if its owner fails to pay the maintenance fees due after 3.5, 7.5 and 11.5 years. Utility patents are by far the most numerous, with more than 90% of the total universe of USPTO patents.[1] According to the Title 35 of the United States Codes (35 USC) section 101: *"Whoever invents or discovers any new and useful process, machine, manufacture, or composition of matter, or any new and useful improvement thereof, may obtain a patent therefor, subject to the conditions and requirements of this title."*[2] In practice however, other types of invention including algorithms can also be patented.[3] The two following sections of the 35 USC defined the condition an invention must meet to be protected by the USPTO: (i) novelty: the claimed invention cannot be already patented or described in a previous publication (35 USC section 102); (ii) obviousness: *"differences between the claimed invention and the prior art must not be such that the claimed invention as a whole would have been obvious before the effective filing date of the claimed invention to a person having ordinary skill in the art to which the claimed invention pertains"*. (35 USC section 103). After review from the USPTO experts, an application satisfying these requirements will be accepted and a patent granted. The average time lag for such a review is on average a little more than 2 years since 1976, with some patents being granted after much more than two years.[4]

[1] Other categories are Plant patents, Design patents and Reissue patents.

[2] Patent laws can be found in http://www.uspto.gov/web/offices/pac/mpep/mpep-9015-appx-l.html#d0e302376

[3] A notable example is the patent *US6285999* protecting the Page Rank algorithm invented by Larry Page in 1998 which was the genesis of Google.

[4] This time lag, sometimes called the grant lag, is highly heterogeneous across technological fields. In addition, it cannot be considered as totally random. For example, if the patent is really disruptive some competitors might have some incentive in delaying the process by disputing the validity of the patent, for more details see [1].

Sample restriction As explained briefly before, we consider every patent granted by the USPTO between 1976 and 2013. For each patent, we gather information on the year of application, the year the patent was granted, the name of the inventors, the name of the assignees and the technological fields in which the patent has been classified (we get back to what these fields are below). We restrict attention to patents applied for before 2007. The choice of the year 2007 is due to the truncation bias: we only want to use information on granted patents and we get rid of all patents that were rejected by the USPTO. However, in order to date them as closely as possible to the date of invention, we use the application date as a reference. As a consequence, as we approach the end of the sample, we only observe a fraction of the patents which have been granted by 2013. Looking at the distribution of time lag between application and grant in the past and assuming that this distribution is complete in time, we can consider that data prior to 2007 are almost complete and that data for 2007 are complete up to 90%.

References

1. Régibeau P, Rockett K. Innovation cycles and learning at the patent office: does the early patent get the delay? Journal of Industrial Economics. 2010;58(2):222–246. Available from: http://EconPapers.repec.org/RePEc:bla:jindec:v:58:y:2010:i:2:p:222-246.
