# Supplementary material for: Classifying patents based on their semantic content
Source: PLoS One. 2017 Apr 26;12(4):e0176310. doi: 10.1371/journal.pone.0176310 (PMC5405987; doi:10.1371/journal.pone.0176310)
Supplement: S2 Text — Detailed description of data collection. (PDF) [file pone.0176310.s002.pdf]

S2 Text : Data collection and Workflow

Data Collection Procedure

Raw version of USPTO redbook with abstracts are available for years 1976-2014 starting from bulk download page at `https://bulkdata.uspto.gov/`. A script first automatically downloads files. Before being automatically processed, a few error in files (corresponding to missing end of records probably due to line dropping during the concatenation of weekly files) had to be corrected manually. Files are then processed with the following filters transforming different format and xml schemes into a uniform dictionary data structure :

- `dat` files (1976-2000): handmade parser
- `xml` files (2001-2012): xml parser, used with different schemas definitions.

Everything is stored into a MongoDB database, which latest dump is available at `http://dx.doi.org/10.7910/DVN/BW3ACK`

Processing Workflow

The source code for the full workflow is available at `https://github.com/JusteRaimbault/PatentsMining`. A simplified shell wrapper is at `Models/fullpipe.sh`. Note that keywords co-occurrence estimation requires a memory amount in $O(N^2)$ (although optimized using dictionaries) and the operation on the full database requires a consequent infrastructure. Launch specifications are the following :

Setup Install the database and required packages.

- Having a running local mongod instance
- mongo host, port, user and password to be configured in `conf/parameters.csv`
- raw data import from gz file : use mongorestore -d redbook -c raw –gzip $FILE

- specific python packages required : pymongo, python-igraph, nltk (with resources punkt, averaged_perceptron_tagger,porter_test)

Running The utility fullpipe.sh launches the successive stages of the processing pipe.

Options *this configuration options can be changed in* `conf/parameters.csv`

- window size in years
- beginning of first window
- beginning of last window
- number of parallel runs
- `kwLimit` : total number of keywords K_W
- `edge_th` : θ_w pre-filtering for memory storage purposes
- `dispth` : θ_c
- `ethunit` : $\theta_w^{(0)}$

Tasks The tasks to be done in order : keywords extraction, relevance estimation, network construction, semantic probas construction, are launched with the following options :

1. `keywords` : extracts keywords
2. `kw-consolidation` : consolidate keywords database (techno disp measure)
3. `raw-network` : estimates relevance, constructs raw network and perform sensitivity analysis
4. `classification` : classify and compute patent probability, keyword measures and patent measures ; here parameters (θ_w, θ_c) can be changed in configuration file.

Classification Data The data resulting from the classification process with parameters used here is available as `csv` files at `http://dx.doi.org/10.7910/DVN/ZULMOY`. Each files are named according to their content (keywords, patent probabilities, patent measures) and the corresponding time window. The format are the following :

- Keywords files : keyword ; community ; termhood times inverse document frequency ; technological concentration ; document frequency ; termhood ; degree ; weighted degree ; betweenness centrality ; closeness centrality ; eigenvector centrality
- Patents measures : patent id ; total number of potential keywords ; number of classified keywords ; same topological measures as for keywords
- Patent probabilities : patent id ; total number of potential keywords ; id of the semantic class ; number of keywords in this class. Probabilities have to be reconstructed by extracting all the lines corresponding to a patent and dividing each count by the total number of classified keywords.

Analysis The results of classification has to be processed for analysis (construction of sparse matrices for efficiency e.g.), following the steps:

- from classification files to R variables with `Semantic/semanalfun.R`
- from csv technological classes to R-formatted sparse Matrix with `Techno/prepareData.R`
- from csv citation file to citation network in R-formatted graph and adjacency sparse matrix with `Citation/constructNW.R`

Analyses are done in `Semantic/semanalysis.R`.
