# Supplementary material for: Classifying patents based on their semantic content
Source: PLoS One. 2017 Apr 26;12(4):e0176310. doi: 10.1371/journal.pone.0176310 (PMC5405987; doi:10.1371/journal.pone.0176310)
Supplement: S3 Text — Extended figures for Network Sensitivity Analysis. (PDF) [file pone.0176310.s003.pdf]

S4 Text : Network Sensitivity Analysis

Network Sensitivity

The example of Fig.1 in main text for a given year yielded the same qualitative behavior for all years, as shown in Fig. 1, 2 and 3 here. We also show an other point of view over the Pareto optimization, that is the third plot giving the values of normalized objectives as a function of θ_c.

Time-window size sensitivity

We show in Fig. 4, 5 and 6 the sensitivity plots used for semantic network construction optimization, for a different time window with $T_0 = 2$. The same qualitative behavior is observed (with different quantitative values, as typically $\theta_w^{(0)}$ is for example expected to vary with document number and semantic regime, thus with window size), what confirms that the method is valid across different time windows.

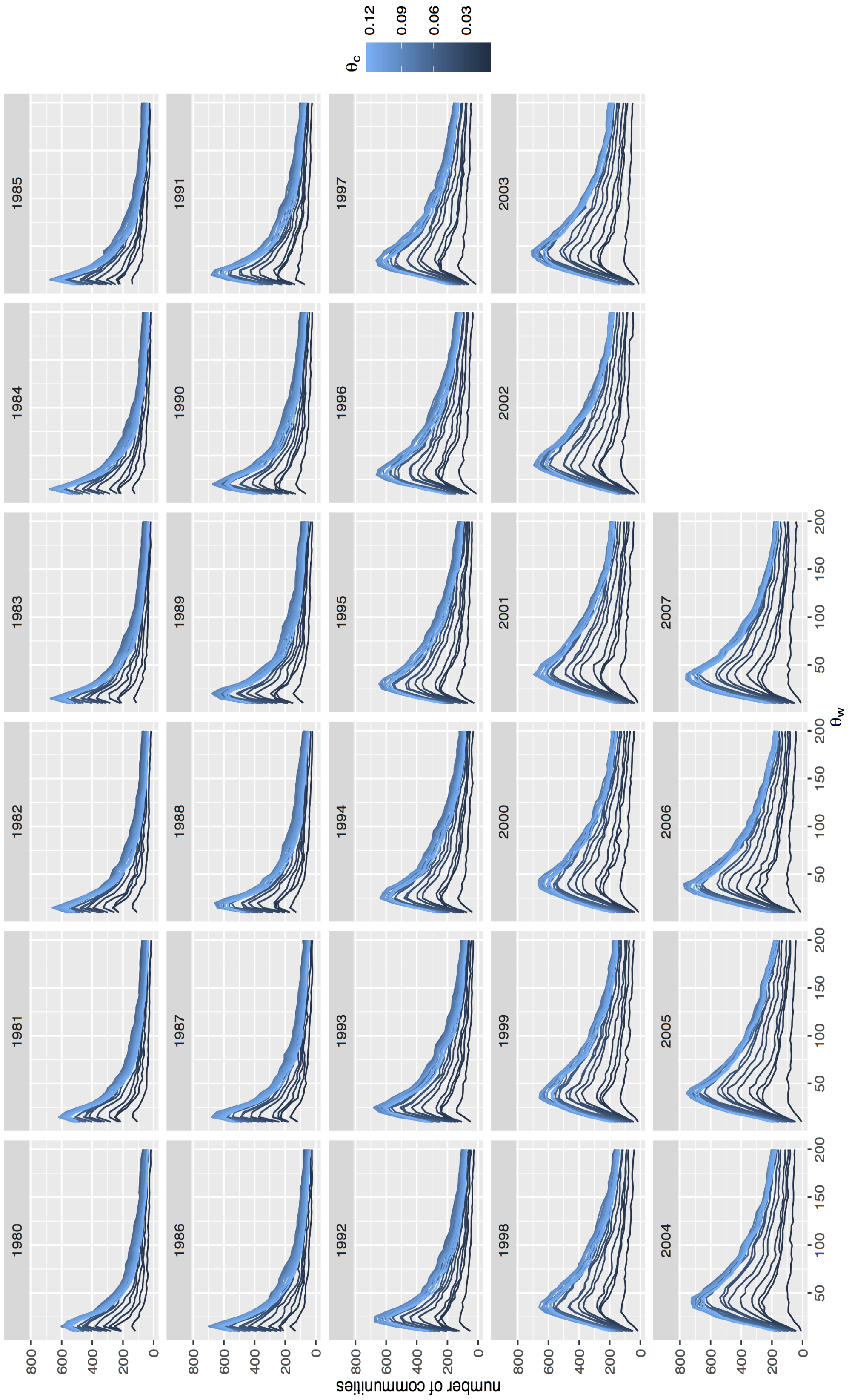

Figure 1. Sensitivity plots for $T_0 = 4$: Number of communities as a function of θ_w, for each year.

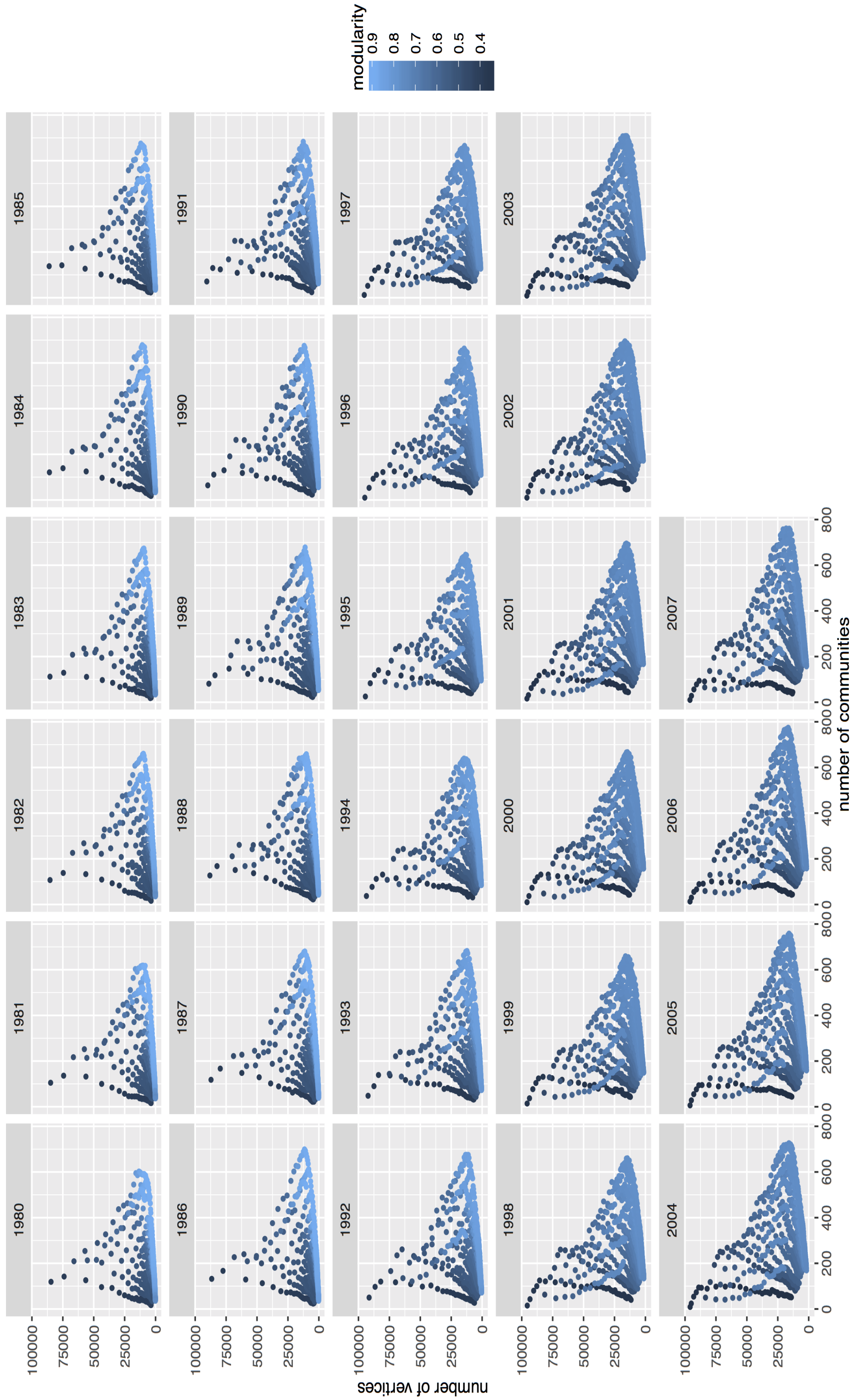

Figure 2. Sensitivity plots for $T_0 = 4$: Pareto plots of number of communities and number of vertices, for each year.

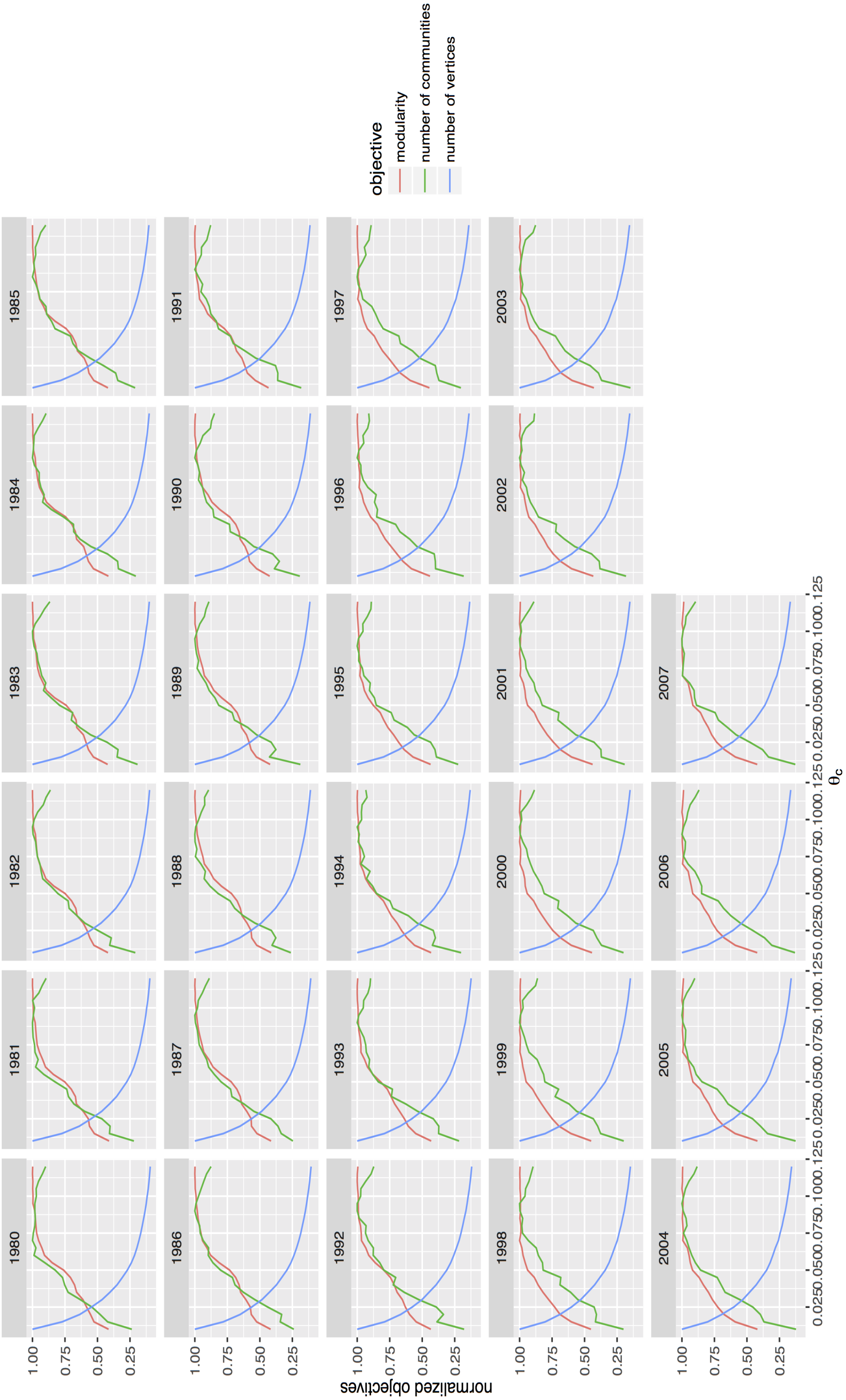

Figure 3. Sensitivity plots for $T_0 = 4$: normalized objective as a function of θ_c, for each year.

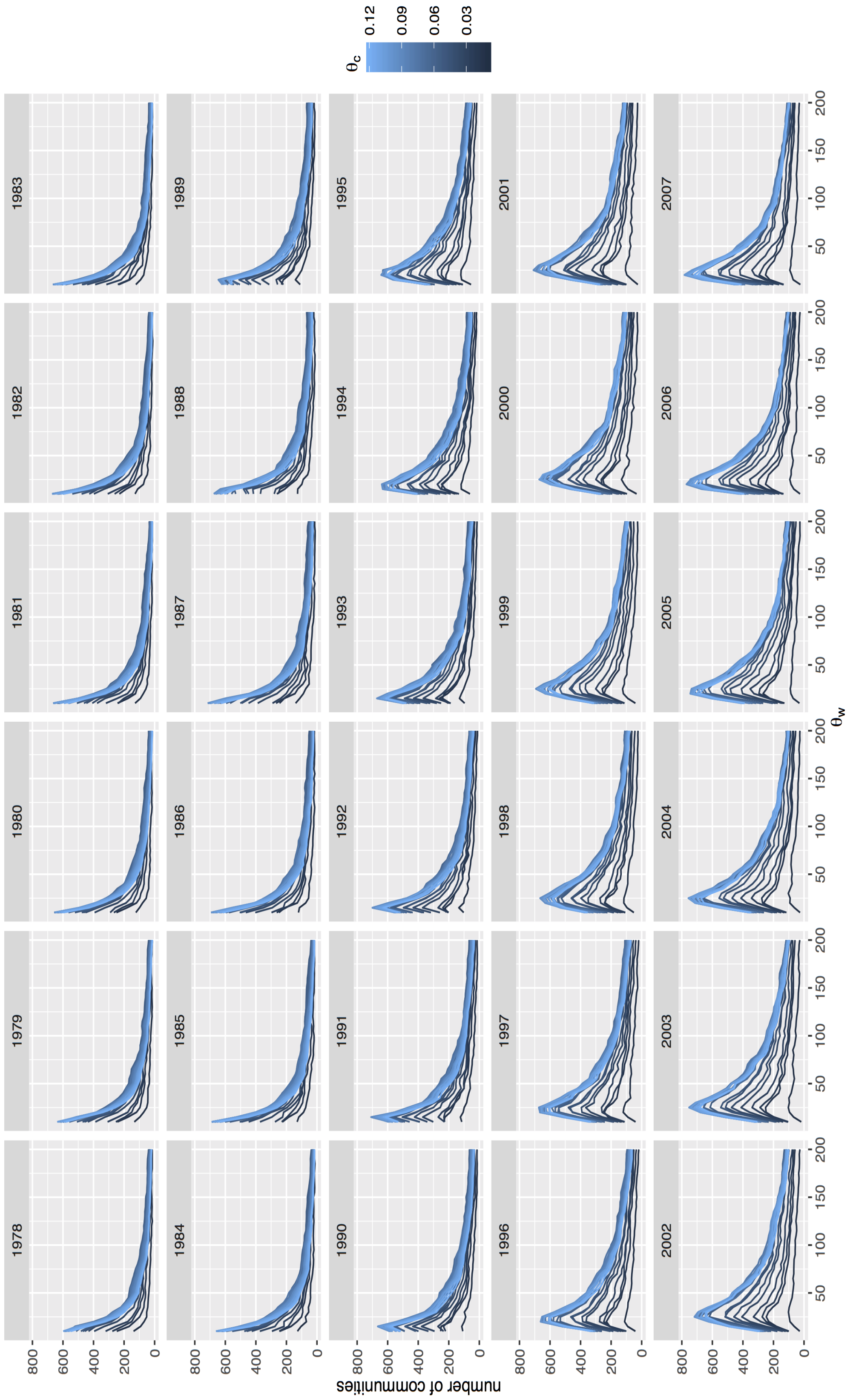

Figure 4. Sensitivity plots for $T_0 = 2$: Number of communities as a function of θ_w, for each year.

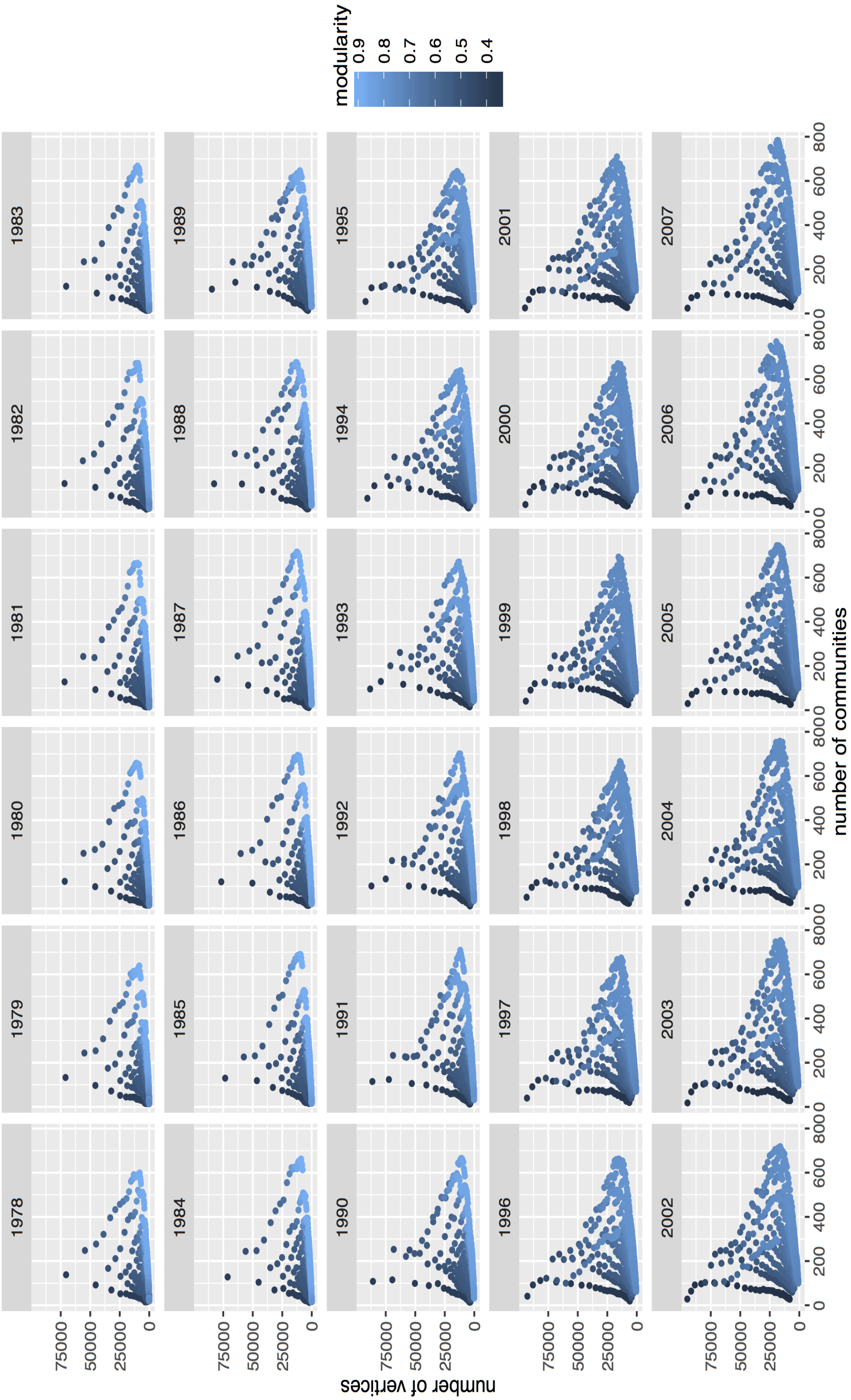

Figure 5. Sensitivity plots for $T_0 = 2$: Pareto plots of number of communities and number of vertices, for each year.

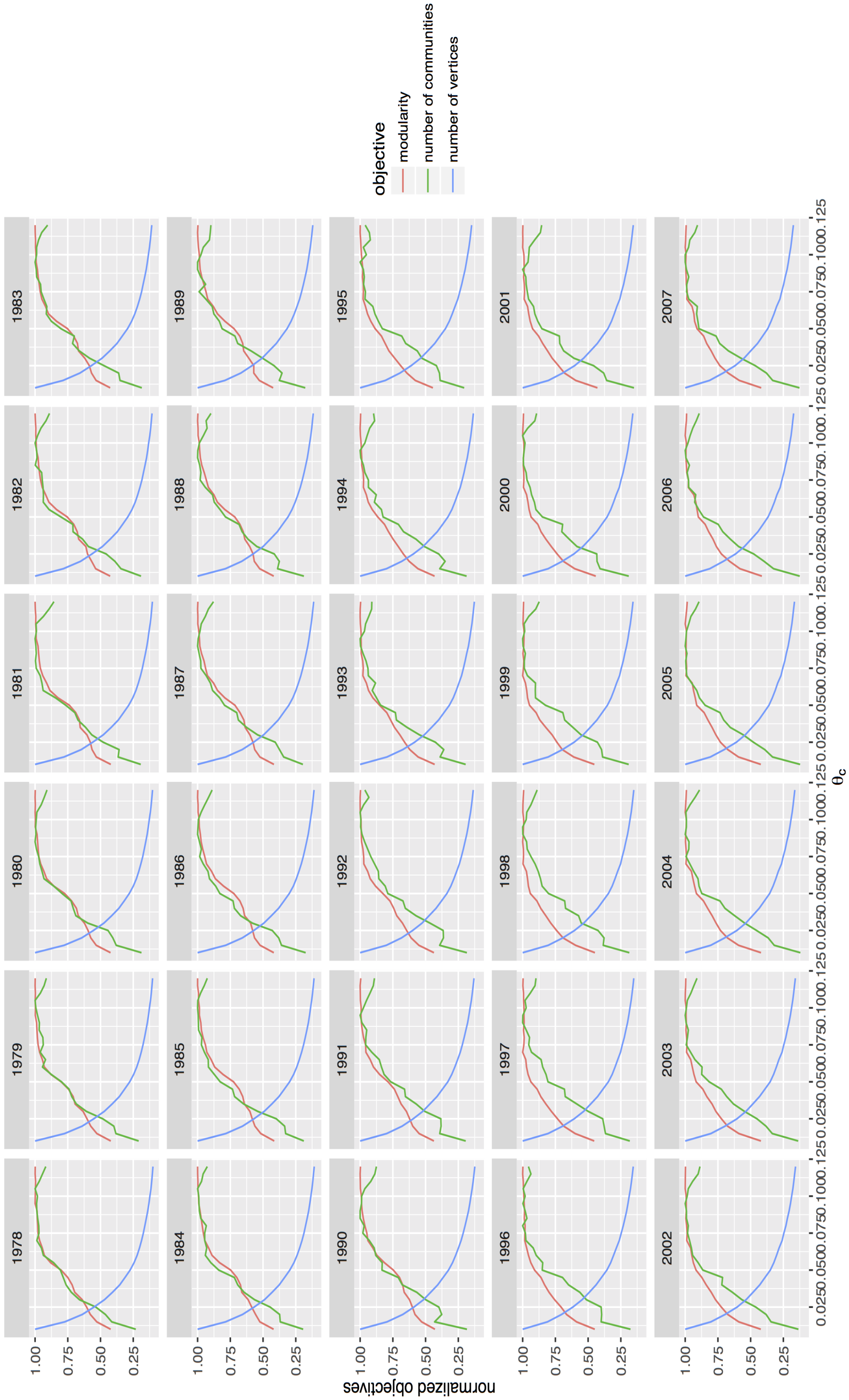

Figure 6. Sensitivity plots for $T_0 = 2$: normalized objective as a function of θ_c, for each year.
