# Supplementary material for: Classifying patents based on their semantic content
Source: PLoS One. 2017 Apr 26;12(4):e0176310. doi: 10.1371/journal.pone.0176310 (PMC5405987; doi:10.1371/journal.pone.0176310)
Supplement: S4 Text — Extended definitions and derivations for the statistical model. (PDF) [file pone.0176310.s004.pdf]

S5 Text : Statistical definitions and derivations

Likelihood expression

We define $\mathcal{F}_i$ the filtration which corresponds to the time t_i. With this notation $\mathcal{L}(X|\mathcal{F}_{i-1})$ simply means the likelihood of X conditioned on the past. We consider $\widehat{\theta}^{(z)}$ the MLE[1] of $\theta^{(z)}$, where the corresponding log-likelihood of the model can be expressed up to constant terms as

$$\sum_{i=2}^{Z}\sum_{l=1}^{C_i}\log\mathcal{L}\big(B_{l,i}|\mathcal{F}_{i-1}\big).$$

Recalling that $B_{l,i}$ are independent of each other and conditioned on the past follow Bernoulli variables

$$B\Big(\min\Big\{1,\frac{N_i^{(z)}}{i-1}+\theta^{(z)}\Big\}\Big),$$

the log-likelihood of the model can be expressed as

$$\sum_{i=2}^{Z}\sum_{l=1}^{C_i}B_{l,i}\log\Big(\min\Big\{1,\frac{N_i^{(z)}}{i-1}+\theta^{(z)}\Big\}\Big)+(1-B_{l,i})\log\Big(1-\min\Big\{1,\frac{N_i^{(z)}}{i-1}+\theta^{(z)}\Big\}\Big). \quad (1)$$

In practice, the user can easily implement the formula (1) for any $0\leq\theta^{(z)}\leq 1$, and maximize it over a predefined grid to obtain $\widehat{\theta}^{(z)}$.

Standard errors of the estimated values

Under some assumptions, it is possible to show the asymptotic normality of $\widehat{\theta}^{(z)}$ and to compute the asymptotic variance. For simplicity of exposition, we assume that we restrict to $\theta^{(z)}$ such that we have $\frac{N_i^{(z)}}{i-1}+\theta^{(z)}<1$ for any $i=2,\cdots,Z$. The central limit theorem can be expressed as

$$\sqrt{Z\mathbb{E}[C]}(\widehat{\theta}^{(z)}-\theta^{(z)})\overset{\mathcal{L}}{\to}MN\Big(0,\int(p+\theta^{(z)})(1-(p+\theta^{(z)}))d\pi^{(z)}(p)\Big), \quad (2)$$

[1] Apparently, this MLE is a partial MLE, but we will not refer to partial for simplicity.

where MN stands for a multinormal distribution and $\pi^{(z)}$ for the asymptotic limit distribution of the quantity $\frac{N_i^{(z)}}{i-1} + \theta^{(z)}$. Note that the variance term in (2) is equal to an aggregate version of the Fisher information matrix. The proof of such statement is beyond the scope of this paper. On the basis of (2), we provide a variance estimator as

$$v^{(z)} = \frac{1}{C_k - 1} \sum_{i=2}^{C_k} \frac{N_{i_k}^{(z)}}{i-1} + \widehat{\theta}^{(z)},$$

where i_k is such that the i_kth patent corresponds to the kth couple. This estimator was used to compute the standard deviation in Table **??**.

Test statistic

The test statistic used is a mean difference test statistic between $\widehat{\theta}^{(tec)}$ and $\widehat{\theta}^{(sem)}$, where the formal expression can be found in (3). We assume independence between both quantities and thus under the null hypothesis, we have that

$$\widehat{\theta}^{(tec)} - \widehat{\theta}^{(sem)} \rightarrow MN(0, V),$$

where $V = \int (p+\theta^{(tec)})(1-(p+\theta^{(tec)}))d\pi^{(tec)}(p) + \int (p+\theta^{(sem)})(1-(p+\theta^{(sem)}))d\pi^{(sem)}(p)$ can be estimated by $\widehat{V} = v^{(sem)} + v^{(tec)}$. Then, we obtain that

$$A = \frac{\widehat{\theta}^{(tec)} - \widehat{\theta}^{(sem)}}{\widehat{V}} \approx \mathcal{N}(0, 1), \tag{3}$$

where A is the mean difference test static.
